# Simple method to generate calibrated synthetic smoke-like atmospheres at microscopic scale

**DOI:** 10.1371/journal.pone.0220700

**Published:** 2019-08-02

**Authors:** Jose M. Nadal-Serrano, Elia Gomez G. de la Pedrosa, Marisa Lopez-Vallejo, Alvaro de Guzmán Fernández González, Carlos Lopez-Barrio

**Affiliations:** 1 ETSI Telecomunicación, Universidad Politécnica de Madrid, Madrid, Spain; 2 Hospital Universitario Ramón y Cajal, Madrid, Spain; University of California San Diego, UNITED STATES

## Abstract

Artificial smokes focusing on macroscopic or fluid properties of smoke have been available for a long time. This paper presents a simple method to generate fully customizable smoke-like atmospheres at microscopic scale (i.e. considering their constituent particles as discrete elements) using a different approach. Synthetic, reproducible media can be generated combining monodisperse microspheres with known geometrical and optical properties conveniently parameterized. The method is presented as a proof-of-concept, highlighting the design decisions along with their implications. Practical issues such as aerosol nebulization, particle carrier selection or the features of the medium chamber where the smoke-like atmosphere is to be tested are analyzed. A comparison between methanol and ethanol as carriers for polystyrene microsphere nebulization is also made. The method could be the seed for the obtention of standard reference media for calibration or standardized characterization of not only smoke detectors and exhaust smoke sensors but also other instruments relying on optical properties of dispersive media (dust in PV panels, public lighting, etc.).

## Introduction

Accurate description of the behavior of smokes and other smoke-like atmospheres is important for a number of disciplines. Just to name a few, in fire safety the assessment and control of smokes is critical to ensure evacuation in case of fire [1–3]. Specially in certain areas of our planet, for instance, it is important to characterize and predict dust clouds as they may be harmful for both infrastructures and people [4, 5]. In that sense, existing commercial solutions that provide with artificial smokes focusing on macroscopic or fluid properties of smoke have been available for a long time. They have been used in manifold fields and applications, from the military to firefighting training and also to validate CFD (Computational Fluid Dynamics) or conduct real-world scale smoke tests [6, 7]. The same can be said for dust clouds or other particles [8, 9].

The increase of environmental awareness at a global scale has prompted the implementation of new policies with strict regulations on greenhouse gases and other emissions [10]. In that sense, the Environmental Protection Agency (EPA) in the US and the European Environment Agency (EEA) in the EU have encouraged innovation including the need for dedicated equipment.

In addition, the fact that in the US deaths from fire and burns are the third leading cause of fatal home injury [11] has led to government-sponsored campaigns promoting the installation of smoke and fire detectors: they have grown as devices critical for safety, not only at homes, but almost everywhere. This ubiquity is closely related to their specificity, which is in turn related to the technology and physics behind the particular process being monitored.

Although regulations and sensitivity testing varies among different countries, regions or vendors, one major obstacle limiting progress in the area is the lack of reference smokes and aerosols for standardized comparison among brand and types of detector or other instruments currently in use to measure the properties of smoke or soot and other particles that constitute smoke and smoke-like atmospheres. In 2012, an expert panel discussed this problem and released a number of recommendations on Soot Standard Reference Materials (SRMs) [12]. Soot formation and aggregation is a very complex process that involves a number of ambient conditions difficult to control at a microscopic level. Several efforts have been made in the past towards the obtention of aerosols that could serve as SRMs using combustion procedures. Some generate them ad-hoc [13], others collect the particles from industrial engines [14]. Dedicated devices have been proposed as well [15], usually aimed at providing a specific percentage of obscuration using aerosol generators [16]. Other authors have followed a different approach: the fabrication of particles with properties assimilable to those of soot (nigrosin, fullerene soot, etc.) [17, 18].

The *main contribution* of this paper is a method to create experimental setups with custom synthetic smoke-like atmospheres generated from a number of parameters: this way the dispersive media is defined at microscopic scale (particles are defined as discrete elements). The method used has been described, along with the specific design issues and their implications towards the fabrication and preparation of the setup for measurements. The method is general enough to hold different particle types. Those types of particles (in fact, their optical properties) would in turn determine the scope and limitations of the media generated, with the corresponding implications in the results obtainable in the experimental setups.

The method presented here as a proof-of-concept opens the door to further research on the generation of, for instance, “reference media” that can be used to calibrate and characterize not only optical smoke detectors or exhaust smoke sensors but also other instruments relying on optical properties of dispersive media (dust in PV panels, public lighting, etc.). The successful generation of reference media (smokes or other smoke-like atmospheres) could have an important impact in the design, certification and comparison between types and brands of detectors, with positive implications for both industry and public safety.

The paper is divided into several parts. The materials and methods section describes the method itself along with the concepts and simplifications made discussing the underlying physics. The preparation and injection of the particulate in the medium chamber is also explained. The results and discussion section gives some insight into the design trade-offs and their implication for experimental setups. As an example, the method and results for the assessment of the most convenient particle carrier is given for polystyrene microspheres. The conclusions and future work lines are stated last.

## Materials and methods

The method designed assumes mainly that smoke and smoke-like atmospheres can be treated as a set of particles. Making so makes it possible to include several types of particles with different properties in the same medium. A smoke model that follows this approach has been shown in [19] and co-works.

The synthetic atmosphere generated following this approach can be fully modeled using a simple set of parameters that characterizes its radiative transfer properties at optical wavelengths:

- The optical properties of each type of particle considered (scattering profile mainly, but also complex refraction index if absorption is to be considered, etc.).
- The statistical distribution of particles according to their type (for instance, size and relative concentration of each type of particle).
- The optical Mean Free Path (MFP) between particles.

It is important to recall that soot particles –and dust, too– have complicated geometries that in the case of smoke even evolve with time. For instance, soot particles are often modeled as fractals [20, 21]. However, when it comes to their optical behavior it is possible to obtain a good approximation using spheres and the Mie theory for a wide variety of cases and a first-order approximation in all cases. In fact, using the *coupled dipole method*, P. Hull et al. [22] were able to validate the Mie model for soot particles (porous clusters of particles with non-spherical symmetry and orientation averaging). Starting with a 100% filled sphere they proceeded to remove spherules at random, obtaining good results when *S*_22_/*S*_11_ did not drop below 0.9. This holds for randomly filled, spherule-filled spheres with a filling of only 20%. The match is better at angles lower than 90°.

Hence, provided that these assumptions hold, it is feasible to model the optical behavior of soot and other smoke-like atmospheres using spherules. Again, where the conditions are not met, a first-order approximation can be still obtained.

In order to consider as well the movement of the particles, the concept of *Aerodynamic Effective Diameter (AED)* is relevant. By definition, two particles will have the same AED if their settling time in a fluid of density 1000kg/m^3^ is the same [23]. The exact value is given by comparison with the time it takes to settle a smooth spherical particle with unit density. Extensive experiments would be needed in this case to obtain AEDs of particles in real-world smoke-line atmospheres.

As a consequence of the previous results, we have used calibrated monodisperse polystyrene microspheres as a convenient material and geometry for the generation of artificial (“synthetic”) smoke-like atmospheres. It is important to recall at this point that the complex refractive index takes into account both the propagation term and the attenuation or absorption term, this last one important when considering soot or black carbon particulate. The complex refractive index of polystyrene [24] is much smaller than that of black carbon [25] (0-0.015 vs. 0.35-0.55). This fact prevents from using the results obtained using polystyrene microspheres for smokes other than smoldering fires. In the case of flaming fires, if absorption is to be considered it would be mandatory to use different materials for soot generation or otherwise consider the effect of absorption.

In the next subsection, the particles chosen along with their preparation is discussed.

Therefore, the effective creation of a stable synthetic smoke-like atmospheres using this approach involves two main steps which will be commented in following sections:

1. Choice and preparation of the synthetic particles.
2. Effective suspension of the particles in a closed chamber suitable for the experiments. This step includes the injection of the particles with a nebulizer.

### Definition and preparation of the synthetic particles

The design of a particular synthetic atmosphere implies the definition of a set of variables. In our case they are mostly reduced to the desired optical mean free path [26] and particle size distribution: the diameter of each particle type considered and its prevalence in%.

The method to ensure that the average mean free path between particles in the generated medium follows the desired value is an important issue and determines the amount of particles in the chamber. In fact, it determines the final amount of each particle type, once the size of the chamber where the synthetic atmosphere will be confined is known. The corresponding calculations are described below.

The geometrical properties of the smoke chamber used for the experimental setup determine its volume and consequently the number of microspheres needed for a target optical mean free path. Proceeding the other way round: the volume fraction of microspheres *ϕ* can be expressed as (following [27, 28]):

$$\begin{array}{c} \phi =\frac{2d}{3Q_{\text{sca}}\Lambda_{\left(MFP\right)}} \end{array}$$ (1)

where *d* is the diameter of the microsphere, Λ_(*MFP*)_ the optical mean free path between microspheres and *Q*_sca_ the scattering efficiency. Focusing on optical applications and using the software provided by Scott Prahl in [29], it is possible to obtain a table containing the values for $\frac{2d}{3Q_{\text{sca}}}$ as a function of the incident wavelength and microsphere diameter; see Table 1.

**Table 1:** $\Psi =\frac{2d}{3Q_{\text{sca}}}$ as a function of wavelength and microsphere diameter.

Lambda vacuum (nm)	*d* = 1*μm*	*d* = 2*μm*	*d* = 3*μm*	*d* = 6*μm*
395	2.24E-007	6.41E-007	8.17E-007	1.99E-006
476	3.61E-007	5.36E-007	8.09E-007	1.76E-006
543	3.20E-007	6.21E-007	9.88E-007	1.81E-006
623	2.23E-007	5.10E-007	7.90E-007	1.75E-006
730	1.66E-007	4.99E-007	1.01E-006	1.67E-006
890	1.50E-007	6.61E-007	7.95E-007	1.73E-006

Naming $\Psi =\frac{2d}{3Q_{\text{sca}}}$ gives the following expression for the volume fraction:

$$\begin{array}{c} \phi =\frac{\Psi }{\Lambda_{\left(MFP\right)}} \end{array}$$ (2)

Eq 2 –and Table 1– make it possible to find the volume fraction of the microspheres as a function of the mean free path in microns.

Once the desired mean free path has been set and the volume fractions computed for the particular size(s) of the microspheres in the experiment, the next step is to decide the volume the chamber, which is the physical space that will hold the synthetic smoke-like atmosphere generated during the experiments. In principle, the bigger the volume, the better, as there is less chance for spurious interactions with the walls of the smoke chamber. Given the high cost of calibrated microspheres, it is necessary to set a convenient chamber volume taking into account the volume fraction of the microspheres and the target experiment.

There are several manufacturers that offer monodisperse microspheres of different sizes for research purposes which met our requirements. Table 2 summarizes the main characteristics of the commercial microspheres used for the experiments.

**Table 2:** Main characteristics of the commercial suspensions of polystyrene microspheres used in the validation experiments.

Manufacturer	Model	Nominal diameter (*μ*m)	Coefficient of Variance (%)	Solid content (%)
Ikerlat Polymers	AJ-1000	1	<0.5	10
Polysciences	19814-15	2	5	2.5
Polysciences	17134-15	3	5	2.5
Sigma-Aldrich	89756-5ML-F	6	<2	2

In our case, 50mm diameter PVC high pressure pipes were chosen for the medium chamber for a number of reasons that will be discussed below. As an example, for an optical mean free path of 100000 *μ*m (10cm) and a 1m long, 50mm diameter pipe illuminated at 395 nm, the amount (volume) of 1 *μ*m microspheres necessary would be:

Vspheres=Vpipe·ϕ=πr2hϕ=π(25·10-3)2·1·ϕ⌋395nm100000μm=

$$\begin{array}{c} =\pi {\left(25\cdot 10^{-3}\right)}^{2}\cdot 2.2410^{-6}=4.4\cdot 10^{-9}m^{3}=4.4\cdot 10^{-3}cm^{3} \end{array}$$

4.4 ⋅ 10^−3^*cm*^3^ of dry polystyrene microspheres, which would correspond to 0.176ml of a commercial 2.5% solid volume suspension.

Once the amount of microspheres to be fed has been calculated following the procedure described above, the next step is to suspend the microspheres in the medium chamber. Next section goes into details on the steps followed.

### Suspension of the particles in a closed chamber

Once the exact amount of each type of particle has been computed –for a certain target synthetic smoke and a given medium chamber–, the next step is to effectively introduce and suspend the particles inside the chamber. For this purpose, a calibrated nebulizer is used to generate an aerosol which will carry the particles to be injected.

It is important to grant that the particles remain stable for the time needed for the experiments and do not aggregate. This last requisite is of particular importance for optical experiments: aggregation would change particle geometries resulting in different scattering profiles and yielding measurements that would not match those expected with the original geometries and optical properties.

The microspheres are all served in aqueous suspensions that contain a surfactant, aimed to prevent aggregation during storage or transportation. It is nevertheless recommended to reduce the time devoted to measurements as much as possible to minimize the possibility of particle aggregation. The method is described in detail in the following sections, along with a study of the most suitable carrier to use.

#### Particle carrier and nebulization evaluation

An aerosol must be generated from the latex containing the microspheres. It is necessary to ensure that the microspheres do not aggregate and that, simultaneously, the aerosol carries them inside the chamber effectively. Once there, the liquid phase of the aerosol (acting as a carrier) must evaporate, leaving the dry homogeneous microspheres suspended.

Water –where the commercial microspheres are originally suspended– is not a good carrier, because the time it takes for water to evaporate is longer than the settling time of the microspheres. Other carriers must be examined, taking into account the wanted features: high volatility and stability, non-solvent of the microspheres used (made of polystyrene in this case) and easy to manipulate. Studying the solvent listing in [30], the candidates are: ethanol and methanol.

In order to assess the best carrier for the microspheres, a set of tests were conducted: the aim was to identify the time intervals where the experimental data could be obtained in the best conditions. The results will be presented and discussed in the Results and Discussion section.

**Fig 1:**
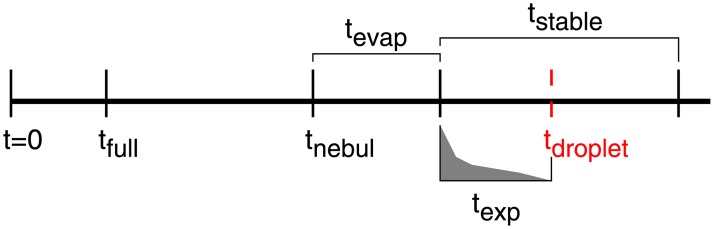
Nebulization process. Time line for nebulization and suitable time interval for the experiments (*t*_exp_, represented in grey). The beginning of *t*_exp_ is the most convenient time frame for measurements.

Fig 1 shows a time line with the most relevant time milestones to be identified during the carrier evaluation experiments. At *t* = 0 the nebulization (injection and aerosolization in the chamber) is started. The aerosol reaches the end of the pipe (chamber) and fills it at *t*_full_. *t*_nebul_ is reached when the solution has been completely nebulized and all the microspheres are in the chamber. The carrier is evaporated *t*_evap_ after *t*_nebul_. From that moment on, the smoke is stable and experiments can be conducted for a period of *t*_stable_. An important moment is where the atmosphere gets saturated and droplets start to appear. This happens at *t*_droplet_ and it is not a desirable situation. Therefore, the time frame to conduct the experiments *t*_exp_ is between *t*_nebul_ + *t*_evap_ and *t*_nebul_ + *t*_evap_ + *t*_droplet_, and the best time frame is right after *t*_nebul_ + *t*_evap_ and as close as possible to this point –the medium evolves (i.e. degrades) unavoidably with time–:

$$\begin{array}{c} t_{\text{exp}}=\{\begin{array}{cc} {}[t_{\text{nebul}}+t_{\text{evap}},t_{\text{nebul}}+t_{\text{evap}}+t_{\text{droplet}}), & \text{if}\;t_{\text{droplet}}\leq t_{\text{nebul}}+t_{\text{evap}}+t_{\text{stable}}\\ {}[t_{\text{nebul}}+t_{\text{evap}},t_{\text{nebul}}+t_{\text{evap}}+t_{\text{stable}}], & \text{otherwise} \end{array} \end{array}$$ (3)

Using the notation above, the ideal aerosol would have the following characteristics:

- Uniform filling.
- Low *t*_nebul_.
- Very short *t*_evap_.
- High stability: long *t*_stable_.
- No droplet formation or very long *t*_droplet_.

Time steps defined above depend largely on the droplet sizes of the aerosol carrying the microspheres as well as on the carrier chosen. The smaller the droplets, the easier it will be (shorter times) to evaporate the carrier. This means that droplets bigger but close to particle sizes are more efficient: the average amount of liquid carrier per droplet to evaporate would be smaller in this case.

Nebulizers that generate calibrated droplets of the size required for the experiment at pressures in the 1-1.5 bar range are not widely available. Nevertheless, medicine nebulizers have been used for years to administer drugs to patients suffering from respiratory diseases [31–33], as drugs contained in droplets in the range of 1-5 *μ*m are known to be effective in reaching the lower part of the respiratory tract [34, 35]. Considering that soot particles generated in compartment fires are in the micron range (with typical diameters for flaming fires around 1 *μ* but extending to over 10 *μ*m) [36, 37], it is possible to use medical nebulizers for a wide variety of smokes and smoke-like atmospheres. In the case of smoldering fires, particles under 1 *μ*m should be considered [38]. A PARI LC nebulizer was used in the experiments conducted.

#### Preparation of the particles

Taking into account that methanol will be used in this case as the preferred carrier –and in general a carrier other than water– it is necessary to make a phase change, from the aqueous solution in which the microspheres are delivered to methanol. Table 2 shows the characteristics of the commercial solutions used, with preparations varying from 2 to 10% solid content.

The phase change procedure is gradual and follows [39, 40]. It is included here for completion:

1. The suspension supplied (100% water) is vortexed.
2. The sample is centrifugated at 11000rpm for 2 minutes.
3. The supernatant is then removed, with caution to avoid removing the microspheres at the bottom.
4. New solvent is added at this point: 75% water, 25% methanol. Steps above are then repeated, adding solvent with increasing methanol concentrations: 50% water, 50% methanol, 25% water, 75% methanol and finally 100% methanol.

Once the suspension in pure methanol has been obtained, it is important to prevent evaporation before the experiment is actually conducted. One option is to use parafilm to seal the Eppendorf wells used with the methanol suspensions and keep them refrigerated at 2-8°C. It is convenient to circumvent the phase change process to the immediate amount needed for the experiments, leaving the rest in water for long term storage (also refrigerated at 2-8°C). It is also important to prevent freezing, as the agglomeration resulting is not reversible by available means (sonication, vortexing, etc.).

A quality control microscope preparation was done prior to nebulization to ensure there was no aggregation.

#### Injection of the particles in the medium chamber

Once the suspension of the microspheres in the appropriate carrier has been obtained, the next step is to actually feed that suspension into the chamber for measuring. The use of PVC high pressure pipes with diameter sizes of 25 and 50mm is convenient, as they provide with enough simulation space when conducting optical experiments using state-of-art sensing devices. Moreover, this form factor retains good maneuverability and a relative low number of microspheres per test –depending on the mean free path of choice–.

Tight control of the relationship between the chamber volume, the aerosol flux entering it and the pressure inside the chamber is necessary to ensure that we do not over pressurize the medium chamber.

In addition, stability is an important issue for the synthetic smoke: its original characteristics must be conserved during the measurements. The use of microfilters has proven useful to avoid overpressure at the chamber and simultaneously retain the particles injected. In the experiments, 47mm MILLIPORE 0.22 *μ*m membrane filters (GSWP 047 00) were used to retain the particles in the medium chamber while letting the excess air out. Moreover, the airflow achieved by this filter helped create and maintain a laminar influx air regime during nebulization.

## Results and discussion

In the previous section, the simple approach followed to create and inject a calibrated synthetic smoke-like atmosphere into a chamber has been described. Using monodisperse microspheres with known size distribution and a predefined optical mean free path it is possible to generate controlled and reproducible smoke-like atmospheres.

The main result and contribution of this paper is the method described itself. However, in this section we will present and discuss the results obtained for the injection process of the particles in the smoke chamber. The experiments conducted to assess the best carrier and time interval to take the measurements will be explained below. A possible experimental setup has been described in detail in [41].

### Synthetic particles, smoke chamber and nebulization process: Design decisions

Polystyrene microspheres exhibit high monodispersity, homogeneity and a uniform spherical shape, which makes them eligible for the generation of smoke-like atmospheres for a number of reasons:

- Monodispersity gives control over the particle sizes present in the experiment. Custom synthetic smoke-like atmospheres with particles of different sizes can be generated building custom particle mixtures that match the desired size distributions.
- In the case of experiments related to optical properties, sphericity makes it possible to compute the scattering profiles directly with high precision using the Mie theory, which gives a first-order approach if analytical computer models are used. Also, they can serve as a good or at least as a first-order approximation to real smokes and smoke-like atmospheres for other applications if microspheres with equivalent effective diameters to those of soot are used. Experiments that consider absorption must take into account the complex refractive index of the microspheres used to evaluate the deviations obtained from the experiments and their impact in real world scenarios.
- Last, polystyrene is a convenient material: it is stable, inert, non-toxic and allows techniques such as vortexing or sonication of the microspheres without damaging or altering their geometrical shape. The spheres must undergo the processes described above to be effectively suspended in the particle chamber.

The choice of the volume and type of smoke chamber is relevant as well. In our case, PVC pipes were chosen for a number of reasons:

- They are black or dark, resulting in reduced reflections of the incoming radiation at their inner surfaces.
- They are round and so they have no corners (except for the endings). This prevents microspheres from being trapped and favors the flow of the medium when being introduced in the chamber.
- They are cheap and easy to manufacture (cut, make openings, etc.).

### Carrier assessment

The measurements to characterize ethanol and methanol in order to evaluate the best carrier for the experiments were made under the following variables:

- Carriers tested for comparison: ethanol and methanol.
- Nebulizers—droplet size: 2, 3 and 7 *μ*m.
- Connectors and pipes: free (no smoke chamber, where applicable), 25mm diameter pipe (400mm and 1000mm length) and 50mm diameter pipe (400mm and 1000mm length).

A transparent PVC pipe was used for the carrier assessment tests, opposing the dark, opaque PVC pipes suggested for optical experiments involving measurements. That way it was possible to see the quality and stability of the whole process. S1 Video shows the injection process. The pipes and the nebulizers were cleaned and dried after each experiment.

The times measured (see Fig 1) are further defined as follows:

- *t*_full_: time elapsed from the beginning of the nebulization to the moment the fog starts to pass through a small aperture left to prevent overpressure at the other end of the pipe. Results are summarized in Table 3. The values shown in the tables correspond to the worst value obtained of the three repetitions that were made for each case.
- *t*_nebul_: time elapsed from the beginning of the nebulization to the moment where no fog is seen coming from the nebulizer for 2 seconds. (Note: when little or very little suspension is left in the nebulizer, nebulization starts to be intermittent. An interval of 2 seconds is a good trade-off between time and amount of nebulized suspension.) Results are presented in Table 4.
- *t*_droplet_: time elapsed from the beginning of the nebulization to the moment when droplets start to appear. They start to appear in the lower part of the pipe, close to the nebulizer inlet. This moment marks the end of an effective nebulization and so, a qualitative result suffices; however the results are available in Table 5. In fact, in all cases *t*_droplet_ < *t*_nebul_ for ethanol, while the contrary, *t*_droplet_ > *t*_nebul_, held true for methanol. This result would justify by itself the use of methanol, as ethanol droplets are formed very early, while methanol did not form any. The explanation could be due to differences in vapor pressure of ethanol as compared to methanol. At 20°C ethanol’s is 5.95 kPa, while methanol’s vapor pressure at the same temperature is 12.8 kPa.
- *t*_evap_: time elapsed from the moment the compressor is stopped (*t*_nebul_) to the moment where the fog cannot be seen. Results are summarized in Table 6.

**Table 3:** *t*_full_ in seconds as a function of droplet size and pipe type.

	Droplet size	Pipe configuration (diameter, length in mm)			
		25⊘, 1000	25⊘, 400	50⊘, 1000	50⊘, 400
**Methanol**	3 *μ*m	3.80	1.50	- (1)	7.06
	4 *μ*m	3.40	1.56	- (1)	5.89
	7 *μ*m	3.50	1.56	15.00	4.98
**Ethanol**	3 *μ*m	3.80	1.70	14.90	4.96
	4 *μ*m	3.40	1.61	14.50	4.48
	7 *μ*m	3.80	1.61	19.20	5.11

(1) Unable to determine. Very faint.

**Table 4:** *t*_nebul_ for 2ml of carrier in minutes:seconds as a function of droplet size and pipe type.

	Droplet size	Pipe configuration (diameter, length in mm)				
		No pipe	25⊘, 1000	25⊘, 400	50⊘, 1000	50⊘, 400
**Methanol**	3 *μ*m	3:35	3:48	3:40	3:25	3:20
	4 *μ*m	3:30	3:45	3:46	3:57	3:49
	7 *μ*m	1:56	1:46	1:49	1:47	1:47
**Ethanol**	3 *μ*m	4:50	5:04	4:52	4:56	4:37
	4 *μ*m	4:28	4:45	4:39	4:50	4:30
	7 *μ*m	2:44	2:45	2:46	2:45	2:47

**Table 5:** *t*_droplet_ in seconds as a function of droplet size and pipe type (‘-’ stands for “not observed”).

	Droplet size	Pipe configuration (diameter, length in mm)			
		25⊘, 1000	25⊘, 400	50⊘, 1000	50⊘, 400
**Methanol**	3 *μ*m	-	-	-	-
	4 *μ*m	-	-	-	-
	7 *μ*m	<10	<15	<20	<20
**Ethanol**	3 *μ*m	-	-	-	-
	4 *μ*m	-	-	-	-
	7 *μ*m	<10	<10	<20	<20

**Table 6:** *t*_evap_ in seconds unless otherwise stated as a function of droplet size and pipe type.

	Droplet size	Pipe configuration (diameter, length in mm)			
		25⊘, 1000	25⊘, 400	50⊘, 1000	50⊘, 400
**Methanol**	3 *μ*m	15	6	16	40
	4 *μ*m	9	7	37	45
	7 *μ*m	15	18	>3 min	40
**Ethanol**	3 *μ*m	>3 min	>3 min	>3 min	>3 min
	4 *μ*m	>3 min	>3 min	>3 min	>3 min
	7 *μ*m	>3 min	>3 min	>3 min	>3 min

An analysis of the data presented in Tables 3 and 6 reveals an important behavioral gap between 2-3 *μ*m and 7 *μ*m particles. Bigger ones have shorter nebulization times due to higher liquid throughput (more volume per sphere). Evaporation times are about the same for all three particle sizes. The only anomaly appears for 7 *μ*m particles in 1000mm, 50mm ⊘ pipes, which exhibit certain fog for longer.

The only worrying result is t_droplet_ for 7 *μ*m particles, as it is shorter than *t*_evap_. Nevertheless, even if droplets are present, for the case of methanol the amount of droplets is very small and does not tend to grow significantly over time. In addition, it is important to recall that this is a worst-case scenario: when introducing the microspheres, the droplets will have particles embedded, accounting for a much smaller amount of carrier to evaporate.

Other techniques such as mildly heating the pipe could prevent drop formation although assessing the potential gain with this technique is left as future work. In that case, temperatures around 50°C (10-15°C below the boiling point) could serve as a good starting point taking into account that the boiling point for methanol is 64.7°C [42]. The results obtained in the different setups support the use of methanol as the most convenient carrier for the injection of the particles.

## Conclusions

In the previous sections, a simple method to generate synthetic smoke-like atmospheres has been presented. Knowing the size distribution of the particles of the desired smoke-like atmosphere and setting the desired optical mean free path between them, it is possible to create custom calibrated atmospheres for a variety of purposes.

The main practical conclusions can be summarized as follows:

- The geometry of the medium chamber and the mean free path condition the volume fraction of synthetic soot particles and ultimately their raw number. Higher volumes, at the expense of increased costs are favored when conducting experiments. Round-shaped geometries are highly convenient. On the contrary, edges and corners can result in undesired turbulence and other unwanted effects during particle injection.
- Possible carriers along with a method to choose the most appropriate one have been studied. Their respective performance have been studied considering their physical properties related to the materials in the microspheres. In the case presented, methanol has raised as the most appropriate one for its superior volatility and easy handling.
- The nebulization process using medical nebulizers has proven fast and reliable. At this point, the use of membrane microfilters has provided with a solution that prevents particles from escaping the chamber while maintaining a laminar air regime during nebulization.

The method presented in this paper can be seen as a good starting point to generate media for the calibration and comparison of optical detectors and other devices: should “normalized synthetic smokes” corresponding to real-world fuels such as paper, oil, wood or other types of smokes were defined, it would be relatively easy to obtain comparable results using this method.

Although the prospective applications of the method presented have focused primarily on smoke, this method can be equally valid to generate other synthetic atmospheres for optical behavior assessment of sensors, PV panels or other devices under urban, desert or other demanding scenarios where smoke-like atmospheres are present.

## Supporting information

S1 Video Synthetic smoke. The whole process of creating and injecting synthetic smoke into the smoke chamber is shown.
(MP4)
